# Imperfect food markets in times of crisis: economic consequences of supply chain disruptions and fragmentation for local market power and urban vulnerability

**DOI:** 10.1007/s12571-020-01084-1

**Published:** 2020-07-16

**Authors:** Rico Ihle, Ofir D. Rubin, Ziv Bar-Nahum, Roel Jongeneel

**Affiliations:** 1 Agricultural Economics and Rural Policy Group, Wageningen University, Hollandseweg 1, Bode 16, 6706 KN Wageningen, The Netherlands; 2 Department of Public Policy and Administration, Guilford Glazer Faculty of Business and Management, Ben-Gurion University of the Negev, Beersheba, Israel; 3 Department of Environmental Economics and Management, The Robert H. Smith Faculty of Agriculture, Food, and Environment, Hebrew University of Jerusalem, Rehovot, Israel; 4 Wageningen Economic Research (WECR) and Agricultural Economics and Rural Policy Group, Senior Scientist & Policy Advisor Agriculture, Environment, and Food, Wageningen University, Wageningen, The Netherlands

**Keywords:** Covid-19, Crisis, Food security, Food supply chains, Oligopolistic food markets, Market power, Resilience, D43, H12, L13, L66, L81, Q11

## Abstract

As these lines were written, the Covid-19 pandemic crisis was continuing to threaten countries around the globe. The worldwide consensus that physical distancing is an effective instrument for mitigating the spread of the virus has led policymakers to temporarily limit the freedom of movement of people between and within countries, cities, and even neighborhoods. These public health-related restrictions on human mobility yielded an unprecedented fragmentation of international and national food distribution systems. Focusing on food retailing - usually being modestly oligopolistic - we take a micro-economic perspective as we analyze the potential consequences this disruption has for the physical as well as for the economic access of households to food at the local level. As the mobility constraints implemented substantially reduced competition, we argue that food retailers might have been tempted to take advantage of the implied fragmentation of economic activity by exploiting their temporarily raised market power at the expense of consumers and farmers. We illustrate our point by providing empirical evidences of rising wholesale-retail as well as farm-retail price margins observed during the Covid-19 crisis. Subsequently, we review existing empirical approaches that can be used to quantify and decompose the micro-economic effects of crises on food demand and supply as well as the size and structure of the market, costs of trade, and economic welfare. The employment of such approaches facilitates policymakers’ understanding of micro-economic effects of public health-induced mobility restrictions on economic activity.

The world is currently undergoing a global pandemic that threatens societies and economies at the global, national, regional, and local levels. Covid-19 appears to be a covariate shock simultaneously challenging many countries of unprecedented magnitude and geographical comprehensiveness unknown in modern history. The livelihoods of millions are challenged by tangible effects as exemplified by the occurrence of extensive hoarding and panic-buying in many Western countries during the first half of March 2020, when most people witnessed empty supermarket shelves for the first time in their lives (The Economist 2020a). Avelino and Hewings (2019) emphasize that we face several challenges in regard to measuring the multifaceted consequences of global disasters. In this paper, we discuss potential economic effects of the fragmentation of economic space as the result of public health restrictions in times of crisis and review empirical techniques suitable for disentangling the adverse micro-economic effects on food demand, supply, and economic welfare.

During early 2020, the international community had witnessed remarkable synchronization of macro-economic effects which had resulted from the pandemic, e.g., in terms of national economic growth and stock markets responses (for an overview see, e.g., The Economist 2020b). These effects were caused by virtually identical challenges to national economies triggered by public health measures recommended by WHO (2020) which were adopted by governments of severely affected countries by and large. National policy responses for mitigating those macro-economic implications of Covid-19 have been similarly synchronous (The Economist 2020c)—the last time such a synchronization of policy actions on a global scale happened during the financial crisis in 2009 (Petrakis et al. 2013).

The synchronization of macro-economic effects is, for instance, visible by the evidence reported by Eurostat (2020a): inflation of the entire consumption basket across the 19 countries forming the euro area fell by more than 40% from February to March 2020 (Table 1) as measured by the Harmonised Index of Consumer Prices (HICP, Eurostat 2018). While prices of the energy component collapsed, the component involving food expenditures was the only sub-category that experienced price increases in comparison with price levels of the same month 1 year ago (+2.4%) as well as for the previous month (+14%). Hence, it seems that the largely synchronized national policy efforts for alleviating the massive public-health threat created a substantial disruptive moment for international food supply chains.

**Table 1:** Average inflation in the euro area in February and March 2020

Inflation in terms of HICP	February 2020 vs. February 2019		March 2020 vs. March 2019		Percentage change from February to March 2020	
All items	+1.2	%	+0.7	%	−42	%
of that:						
Food, alcohol & tobacco	+2.1	%	+2.4	%	+14	%
Services	+1.6	%	+1.3	%	−19	%
Non-energy industrial goods	+0.5	%	+0.5	%	0	%
Energy	−0.3	%	−4.3	%	−1333	%

Source: Authors’ calculations based on Eurostat (2020a)

Notes: Details on the construction of the HICP can be found in Eurostat (2018)

Food-security related effects of such disruptions differ in magnitude depending on whether food consumption of most households is largely met by subsistence farming or gardening, a system which prevails in many rural areas worldwide, or whether the food system relies on the market mechanism whose functioning is crucially determined by the resilience of international food supply chains (Ansah et al. 2019). The cities and regions hit hardest by Covid-19 by the end of April 2020 all belong to the latter type and are characterized by: 1) well-functioning food markets that are based on complex and highly specialized food supply chains, which often cross several national borders and 2) subsistence food production playing only an insignificant role in their national food provision. Examples are Wuhan, a city of 8 million inhabitants; Lombardy, a region with the second highest population density in all of Italy; Madrid; and New York City.

The stability of food supply chains which are reliant on the market mechanism comes often hand in hand with the emergence of locally concentrated retailing structures, in which the embracement of an environment of low competition (Apergis and Polemis 2015) is considered an acceptable side-effect of maintaining stable and health-regulated supply channels that offer a continuous and broad portfolio of fresh and processed food commodities. The typical oligopolistic structure of only a handful of dominating supermarket chains per nation^1^ is usually tolerated since the supply from international food markets is fairly competitive, thereby keeping consumer prices at reasonable levels. These supply chains have helped to deliver the high standards of living found in Western countries by pushing down households’ food expenditures to about one eighth of total expenditures (Eurostat 2019).

The quick spread of the pandemic shakes this status quo in local food markets with respect to two major aspects, both of which are particularly important in the context of urban conglomerations that have very high population densities and, therefore, rely on market-based food systems. First, in the absence of significant subsistence production it is crucial to maintain continuous food trade between as well as within countries in times of crisis. During periods when international food supply chains and regional and local markets are temporarily fragmented due to closures of national or regional borders, maintaining usual levels of food supply becomes severely challenged and is likely to yield higher retail prices at the local level. Such a rise in retail prices may be driven by either scarcity resulting from delayed and fewer deliveries - processing plants might have needed to shut down - or increased transaction costs. These costs may rise as a result of logistics personnel getting infected by the pandemic, distribution processes for home delivery having to be newly set up, or lorries needing to wait much longer at border crossings, etc. Most importantly, the implementation of hygiene-related crisis-mitigation measures at the firm level such as installing plastic protectors, retraining staff, extended shop opening times, e.g., for vulnerable parts of the population, or a reduction in overall demand as only limited numbers of people were admitted to shops might have required retailers to spread fixed costs over fewer products sold and, thus, resulted in higher consumer prices. For farmers, transport and supply chain disruptions result in substantial temporary plunges in, for example, availability of seasonal farm labor and market demand for their produce. The more perishable the primary produce is, the more pronounced will be the resulting economic effects. Their range stretches from delicate drops in wholesale or farm-gate prices to complete market collapses as, for example, reported by The New York Times (2020) for the cut flowers market.

The second aspect relates to consumers’ physical access to food at the local level and their ability to choose from a sufficiently large portfolio of competing offers. One of the major national policy measures hastily legislated in nearly all affected countries has been severely limiting the freedom of movement of consumers in order to mitigate the public health threat of Covid-19. This synchronization of policy response is based on the worldwide consensus, which follows the recommendations of the WHO (2020), that physical distancing of potential human disease vectors is a key tool for slowing down the tearing pace of the spread of this virus. Implementing this measure resulted in comprehensive constraints on human movement between and within countries as well as complete lockdowns in areas hit by high infection rates. Countries closed their borders to non-resident travelers. The geographical scope citizens were allowed to reach within their region, city or neighborhood was suddenly massively constrained. Leaving the own residence has often been restricted to only the most essential purposes. In this way, these restrictions on human mobility added another temporary layer of fragmentation of economic space at various spatial and institutional levels to the already existing socio-environmental fragmentation of urbanized regions (Link et al. 2014).

This fragmentation has the potential to severely impair all four dimensions of food security (FAO 2008) at the local level. First, the legal limitations that reduce the usual portfolio of market outlets for food purchase severely restrain physical access to food by cutting off many of these outlets from the reach of customers because either they are located beyond the permitted thresholds of individual movement or they needed to temporarily shut down. Many businesses selling non-essential commodities or those requiring the physical proximity of customers, such as restaurants or cafés, have been ordered to close for several weeks in order to minimize physical human interaction. These restrictions imply that consumers’ possibility to compare quality and prices between competing shops and reach the one giving them most utility,^2^ even if the shop is located at the other end of town, becomes temporarily severely constrained.

This effect is further magnified the more geographically limited consumers’ mobility becomes and the more competing retail businesses - many of them engaged in food services or the sale of processed food - need to temporarily shut down based on the emergency legislation. The tighter these restrictions are, the more consumers will be prevented from visiting their habitual food retailers and food services providers. For example, inhabitants of Moscow have been legally obliged to only visit the grocery store closest to their place of residence (Sobyanin 2020). Such restrictions force consumers to substitute within their reduced portfolio of food suppliers and, therefore, may lead them to adapt their dietary composition as well as contribute to the broadly observed hoarding. The many empty supermarket shelves (The Economist 2020a) attest to the difficulties that the food outlets allowed to remain in business faced when trying to ensure permanent availability of food at the local level.

Such limited physical availability and access are likely to translate into challenges for the economic access to food as well. Household income might suddenly fall short of habitual levels due to temporary unpaid employer shutdown or permanent unemployment. Retail outlets such as supermarkets or food pickup shops, delivery services or drive-throughs that continue to open for being visited by clients might be tempted to take advantage of their temporarily enhanced oligopolistic power resulting from the reduced number of operating and accessible food retailing outlets at the local level. Likewise, they might seize the opportunity to exploit a comparably resulting oligopsonistic situation in their input markets by pushing down purchase prices.^3^

Hence, core measures implemented to relieve this synchronous public health crisis of extraordinary magnitude and very rare incidence have the potential to severely impair economic activity at the local level by yielding temporary increases of market power of businesses that maintain food provision. The economic fragmentation being currently experienced in many Western countries enables these businesses to realize additional markups by raising retail prices, pushing down wholesale prices, trying to capture margins of competitors whose operations are temporarily banned, or overcharging fees for home delivery, for example. This behavior is likely to be visible in retail price increases of food and, thus, higher than usual food expenditures (Table 1), despite of camouflage efforts potentially employed by sellers (Ferguson 2014).

The aggregated magnitude at the national level of such irregular food providers’ behavior can become substantial and threaten food security and the nutritional status, especially for low-income households. This effect becomes magnified as many food banks commonly existing in many market-reliant food systems had to temporarily stop operations since they typically involve extensive direct physical human interaction. Reduced dietary quality in combination with the enforced short-run substitution of food consumption patterns may, in turn, make individuals more susceptible to the pandemic and other health-related risks. Hence, it is essential for policymakers to gain an understanding of the potentially complementary micro-economic effects of this disruption of economic activity on food security.

Next, we provide examples of price changes in two market-reliant food systems – the EU as well as Israel being a non-European country which has a very high population density of about 400 people/ km^2^ – illustrating our argument that retail prices experienced a pronounced increase during the time when mobility limitations were in place. Figure 1 visualizes the trajectories according to which consumer and producer price indices for all food commodities in the EU changed from February to April relatively to their January values of 2019 and of 2020, respectively. The connected scatterplots (Haroz et al. 2016; Acosta et al. 2020) indicate that changes from January to February in both years have been very similar. However, for March and especially April the trajectories of both indices – and, therefore, also the trajectory of the margin between average retail and farm-gate prices – strongly differ between both years. While the food producer price index had increased in April 2019 much more than the food consumer price index, was it the food consumer price index in April 2020 which had skyrocketed in comparison to its value at the beginning of this year whereas the food producer price index had barely changed. Hence, food markets in the EU had experienced a squeezed margin between average retail and farm-gate prices in 2019. One year later, this margin was heavily stretched when comprehensive restrictions on individual mobility were enforced throughout the EU.

**Fig. 1:**
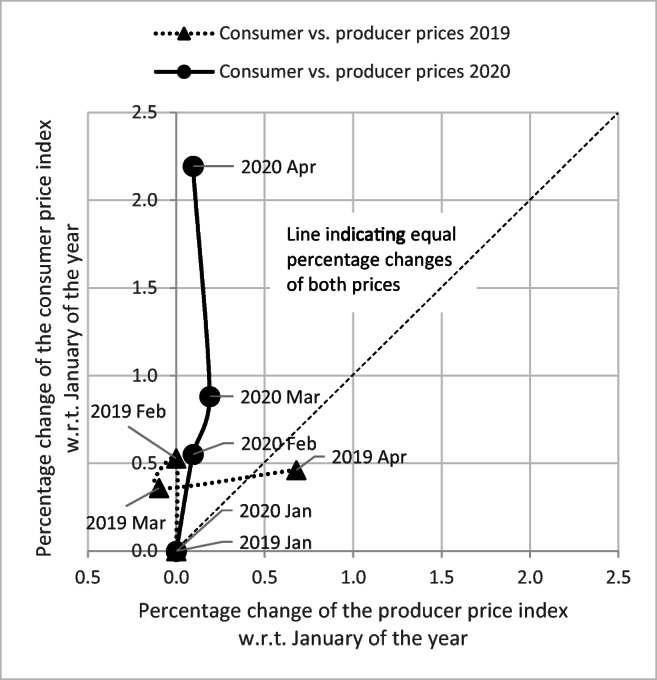
Changes in average producer and consumer prices of food in the EU. Notes: Both price indices are harmonized averages over comprehensive baskets of food commodies for the entire EU27 (without Great Britain). For details, see the metadata available in Eurostat (2020b). Mobility restrictions due to Covid-19 started in most EU countries around the middle of March and remained in place and were often tightened in April 2020. The dashed line indicates equal percentage changes of both prices, that is, a constant percentage margin between them. All points above (below) the dashed line denote changes in both price indices which enlarged (diminished) the percentage margin between average retail and farm-gate prices. The more the orthogonal distance of a point to the dashed line, the more unequal the changes in the indices in the respective month. The two curved lines connect observations of subsequent months and thus allow a visual impression of how both price indices changed in February to April in comparison to their respective January values in each year. See Haroz et al. (2016) for details on how to read connected scatterplots and Acosta et al. (2020) for an example how they can be used for explorative analysis.

The second example, presented in Table 2, displays the change in retailers’ margins of selected fresh fruits and vegetables that have a principal role in the Israeli diet (Israeli Ministry of Agriculture and Rural Development 2019b). In comparison with the weeks prior to the start of the economic fragmentation due to legislated national distancing restrictions, retail-wholesale-price margins rose substantially for several commodities presented in Table 2. Decomposing these margin changes illustrates that retail prices increased for all commodities. Sizable reductions in wholesale prices enlarged retailers’ margins for selected commodities, providing prima facie evidence that the growth in retailers’ margins took place at the expense of consumers and producers alike. The fifth column of Table 2 reports the changes in average retailers’ margins for the identical week numbers for the preceding year highlighting that the margins in 2020 showed a distinct pattern in comparison with the previous year as average margins of these commodities increased less or even shrank during the same months of 2019.

**Table 2:** Changes in average retail-wholesale-price margins before and during Covid-19 in Israel

Commodity	Changes in average margins during the Covid-19 period		Contribution of retail price change		Contribution of wholesale price change		Changes in average margins during the 2019 benchmark period
Tomatoes	+505	%	+209	%	−296	%	−217%
Avocados	+62	%	+7	%	−55	%	+49%
Apples	+28	%	+23	%	−5	%	−17%
Cucumbers	+10	%	+98	%	+88	%	−120%

Source: Authors’ calculations based on Israeli Ministry of Agriculture and Rural Development (2020)

Notes: These commodities where chosen as they play a significant role in the average Israeli diet and thus are subject to similarly high and continuous food demand and, therefore, food and nutrition security in the country. The margins reported in the second column refer to the difference between the retail and wholesale price measured in New Israeli Shekels (NIS)/kg. The third and fourth columns split the change reported in the second column into the contributions of the retail price *p*^*r*^ as well as the wholesale price *p*^*w*^, respectively, according to:
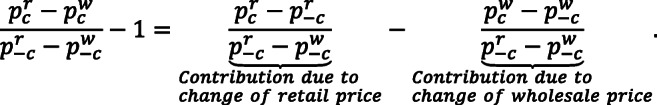

All weekly observations until end of week 14 of 2020 have been considered. The Covid-19 period (denoted as “c” in the preceding formula) is defined by the start of the considerable physical distancing restrictions at the national level in Israel, such as the closing of the entire education system, at the beginning of week 11 of 2020. The benchmark margins in the pre-Covid-19 period (denoted as “-c” in the preceding formula) are the averages of the first ten weeks of 2020 before the start of the restrictions. The 2019 benchmark period in the fifth column refers to the changes in average margins during the same week numbers in 2019 corresponding to the Covid-19 period and the pre-Covid-19 period in 2020. Any other potential price determinants on the demand as well as on the supply side such as demand shifts, seasonality effects, adverse weather conditions, or pests/locust invasions did not change between these two periods to the best of our knowledge. The only determinant which changed between these two periods were the considerable physical distancing restrictions at the national level. Therefore, the observed change in the margins can be plausibly attributed to Covid-19.

The different approaches amassed by economic literature to identify and measure price-cost margins have proven to be useful in many irregular market contexts (Appelbaum 1982; Bresnahan 1989). Examples of such contexts include the automobile industry (Berry et al. 1995), the electricity generation industry (Klemperer and Meyer 1989), the banking sector (Molyneux et al. 1994), and food markets (Genesove and Mullin 1998; Nevo 2001; McManus 2007; Villas-Boas 2007). While these empirical tools became the workhorse for antitrust authorities,^4^ they have barely been utilized to understand the effects of crises. With modest adjustments, these models can be harnessed to decompose micro-economic consequences of crises such as Covid-19.

For the empirical quantification of micro-economic effects of crises, there are just a few examples in the literature. Growitsch et al. (2014) used a Cournot oligopoly model to assess the effects of disruptions in the liquefied natural gas supply chain. They exploited hypothetical blockages of the Strait of Hormuz as supply shocks in a spatially oligopolistic context. Moch (2013) studied the behavior of the fragmented banking sector of Germany during the subprime crisis. He used the Panzar and Rosse (1987) revenue approach,^5^ in which the competition level in separate markets is evaluated based on the monopolistic profit maximization rule. Moch (2013) showed that measuring competition at an average country level does not provide suitable assessment of fragmented markets. European Commission (2020) used a micro-simulation based on farm accountancy data to assess short-run effects of crisis shocks on farm liquidity.

A class of tools that can be used to separate the measurement of potential consequences of temporary fragmentation of food markets at the micro-economic level are differentiated goods oligopoly models (Berry et al. 1995). These models simultaneously account for the variation in prices and market shares of various versions of commodities, for example, brands or varieties. Commodity prices are modelled as a function of observable and non-observable cost indicators, the markup that stakeholders in an oligopolistic industry are able to realize, and potentially a set of variables that specify institutional market characteristics. Market shares are modelled as functions of observable and non-observable product characteristics, product prices, and potentially differing sets of institutional market characteristics.

Such models are capable of decomposing the effects of market crises into four dimensions: demand, supply, market structure, and costs of trade. For example, Fershtman and Gandal (1998) measured the effect of a politically enforced market fragmentation in the form of the boycott by Arab countries of the automobile market of Israel. While taking into account the oligopolistic structure of car retailing, they separately quantify the effects of the discontinued embargo on the Israeli automobile industry on market prices and changes in consumer surplus. Bar-Nahum et al. (2020) added a fifth dimension to the analysis by allowing to estimate a supposed change in market size due to temporary fragmentation of economic activity caused by escalations of violent political conflict. This framework can be adapted for separating the micro-economic effects of any major unexpected crisis, such as Covid-19, that potentially temporarily reduce physical access to markets.

The abovementioned methodologies can be useful to measure complementary aspects of the effects resulting from imperfect competition in (temporarily) fragmented food retail markets. Oligopoly models can be adjusted to verify whether and in which ways the transiently improved bargaining positions of selected players in food markets enlarge crisis ramifications. Demand planning (Swierczek 2020) and disruption management emergency plans (Chakraborty and Sarmah 2019) at the national or trans-national level can be a feasible tool for anticipating and minimizing the effects of potential future covariate shocks of the magnitude of Covid-19 on food markets and food security. Existing food price monitoring schemes (Baltussen et al. 2019; Eurostat 2020b) can be directly adapted (e.g., FAO 2020) to serve policymakers as live monitoring tools that identify noncompetitive pricing behavior during times of crises.

## Footnotes

1 McCorriston (2002) and Swinnen and Vandeplas (2010) report concentration estimates in historical perspective. Returns on investment that can be considered to be a measure of oligopolistic profits in food retailing are typically within the range of 5 to 10% (Sellers-Rubio and Más-Ruiz 2009; Israeli Ministry of Agriculture and Rural Development 2019a).

2 “In the language of economics, the concept of utility refers to the numerical score representing the satisfaction that a consumer gets from a market basket.” (Pindyck and Rubinfeld 2001, p. 73)

3 This economic phenomenon is closely linked with legal and historical questions relating to antitrust issues in the area of food supply chains (Hawk 2018) and ethical questions regarding price gouging in the context of food (Zwolinski 2008; Snyder 2009). Reduced local competition in food retailing is furthermore closely connected to currently ongoing public discourses about the fairness of food prices (Xia et al. 2004; Ferguson 2014) and the issue of unfair trading practices in food supply chains (Schebesta et al. 2018) which recently gained substantial political momentum (Council of the European Union 2019). All these topics are beyond the scope of this paper but provide ample scope for future research for understanding legal and ethical implications of Covid-19.

4 See Digal and Ahmadi-Esfahani (2002) and Cotterill (2006) for early reviews.

5 Bikker et al. (2012) provide a comprehensive critical reading and application of the Panzar-Rosse revenue approach.
